# Alert burden when monitoring patients’ vital signs continuously at home

**DOI:** 10.1007/s10877-026-01465-5

**Published:** 2026-07-03

**Authors:** Emilie Sigvardt, Jesper Mølgaard, Hanne Nygaard, Christian S. Meyhoff, Eske K. Aasvang

**Affiliations:** 1 Department of Anaesthesia and Intensive Care, Copenhagen University Hospital – Bispebjerg and Frederiksberg, Bispebjerg Bakke 23, Copenhagen, 2400 NV Denmark; 2 Department of Anesthesiology, Center for Cancer and Organ Diseases, Copenhagen University Hospital – Rigshospitalet, Copenhagen, Denmark; 3 Department of Emergency Medicine, Copenhagen University Hospital – Bispebjerg and Frederiksberg Hospital, Copenhagen, Denmark; 4 Department of Clinical Medicine, University of Copenhagen, Copenhagen, Denmark

**Keywords:** Vital signs, Continuous monitoring, Remote monitoring, Vital signs monitoring

## Abstract

Continuous monitoring of vital signs after hospital discharge may support early recognition of deviating vital signs. However, the utility may be challenged by high alert frequencies. This exploratory study aimed to assess the impact of evidence-based augmented filtering algorithms on alert frequency following discharge. Adult patients (≥ 18 years) discharged after acute medical admission were monitored continuously using wearable devices that measured heart rate, respiratory rate, blood pressure, and oxygen saturation. The primary outcome was the number of alerts per patient per day. We compared outcomes across three filtering strategies: (1) no filtering, (2) artefact removal, and (3) filtering with artefact removal and clinical criteria based upon severity and duration. Ninety-eight patients were enrolled; the total vital sign alert frequency was reduced from a median of 74 [IQR 36–125] to 5 [IQR 1–13] alerts/patient/day following application of the clinical criteria filters, corresponding to an 84% reduction (*p* < 0.001). Alert frequency following the three filtering approaches was 74 [IQR 36–125], 67 [IQR 33–103], and 5 [IQR 1–13] alerts/patient/day, respectively, *p* < 0.001. Artefact removal and the application of filters based on severity and event duration significantly reduced alert frequency in patients continuously monitored at home after hospital discharge. Further studies are needed to evaluate clinical safety and predictive value.

**Supplementary Information:**

The online version contains supplementary material available at https://doi.org/10.1007/s10877-026-01465-5.

## Introduction

Continuous monitoring of vital signs in hospitalized patients outperforms manual intermittent monitoring regarding the detection of vital sign deviations, and a significant reduction in adverse events and healthcare costs has been documented [1–3]. Home monitoring of recently discharged patients is emerging as a relevant extension of hospital care, offering the possibility of similar timely recognition of post-discharge deterioration and avoidance of preventable readmissions [4, 5].

Continuous vital sign monitoring with wearable devices provides clinicians with real-time data on patients’ physiological status and can trigger alerts in response to abnormal vital signs [2, 6]. However, alert fatigue is a well-recognized barrier to the effective use of continuous monitoring systems [7]. Studies from intensive care units have shown that up to 80–90% of alerts are non-actionable, leading to desensitization among clinical staff and delayed responses to true deterioration [8]. This highlights the importance of designing alerting systems that detect true deterioration without generating excessive noise.

Monitoring in home settings is technically feasible [9] but differs fundamentally from the hospital environment: patients are typically more mobile, interact less with healthcare personnel, demonstrate greater physiological variability due to daily activities, and have different adherence to wearing the devices [10]. These factors may contribute to an increased likelihood of data artefacts and false alerts when patients’ vital signs are monitored remotely [11]. Without appropriate filtering, continuous vital sign monitoring at home carries an inherent risk of generating large volumes of non-actionable alerts, placing unnecessary burden on the attending healthcare provider [6, 9, 12].

Various strategies - such as artefact removal, adaptive thresholds, and delay algorithms - have been developed to reduce alert burden, but these have primarily been designed and validated in in-hospital settings [13, 14], and there is limited evidence to support their applicability in patients recovering at home. Existing studies are based on data from healthy volunteers or simulated scenarios [15–17]; hence, it cannot be assumed that alert dynamics effective in hospitals will perform similarly in the home. This underscores the need to investigate such strategies specifically in the home setting.

This exploratory study aimed to assess the impact of evidence-based augmented algorithms on alert frequency following hospital discharge. We hypothesized that application of these algorithms would reduce alert frequency compared with both unfiltered data and data with artefact removal.

## Materials and methods

### Study design

This was an explorative cohort study analysing data from two prospective studies (NCT05223504 and NCT05536206). Both studies were part of the Wireless Assessment of Respiratory and circulatory Distress at Home (WARD-HOME) project. All eligible patients provided written informed consent before enrolment. The studies were approved by the regional ethics committee (H-20009132, including subsequent amendment 91724) before data collection and analysis. This sub-analysis was registered at ClinicalTrials.gov (NCT07096648) before data extraction.

### Setting

Patient recruitment was conducted between August 2021 and July 2023 at Bispebjerg Hospital, Copenhagen, Denmark. Overall inclusion criteria were adult patients (≥ 18 years) admitted with an acute medical disease and scheduled for discharge within five days from inclusion. Exclusion criteria were allergy to plaster/silicone, pacemaker or implantable cardioverter defibrillator device, inability to give informed consent, or deemed not able to open the front door when visited by the investigator.

Patients were enrolled in the studies during the final days of hospitalization, and the study period was defined as starting when patients entered their home environment. All patients in our study adhered to standard discharge criteria and were not discharged earlier due to study participation.

Up to three wearable and wireless monitoring devices were applied to the patient either before discharge or in the patient’s home shortly thereafter, with the intent of a maximum of eight days of monitoring. Patients were taught how to replace the devices following personal hygiene and how to change batteries after two days of monitoring. Daily follow-up, either in-person or by telephone, was conducted to ensure correct device placement, assess signal quality, and confirm patient well-being. Known device error values and physiologically implausible readings were excluded before analysis. Because blood pressure is measured less frequently than continuously monitored vital signs, missing blood pressure values were carried forward for up to 60 min to avoid underestimating alert rates. Oxygen saturation was summarised as the highest value recorded within each 5-minute window to reduce the influence of movement artefacts. Monitoring periods with fewer than 6 h of valid data per day were excluded from the analysis.

### Variables

Demographic data were obtained from the electronic medical record and included height, weight, former and current diagnoses, and smoking history. Continuous monitoring of vital signs was performed using up to three CE- and FDA-approved devices measuring heart rate (HR), respiratory rate (RR), systolic and diastolic blood pressure (SBP and DBP), and peripheral oxygen saturation (SpO₂). All devices were connected via Bluetooth to a gateway tablet (Isansys Lifecare, Oxfordshire, United Kingdom), which transmitted data through an encrypted 4G connection to the hospital’s secure internal server. HR and RR were measured using a single-lead electrocardiogram (ECG) patch (Lifetouch, Isansys, Oxfordshire, United Kingdom), which recorded ECG signals for detection of QRS complexes and R peaks. The ECG was only transmitted every 10. second to reduce battery consumption of the devices. SpO₂ was measured using a wrist-worn pulse oximeter (WristOx 3150, Nonin Medical Inc., Minnesota, USA), capturing SpO₂ values with each pulse beat. SBP and DBP were measured using an automated cuff-based device (TM-2441, A&D Medical, Tokyo, Japan) at 60-minute intervals. Patient outcomes were assessed from the electronic medical record 30 days after discharge to identify any serious adverse events (SAEs) occurring in the period following hospitalization. All adverse events were classified according to a predefined outcome manual that specified a total of 35 outcomes, grouped into eight different categories: neurologic, respiratory, cardiovascular, infectious, other complications, mortality, ICU-admission, and readmissions.

### Exposure variables

The exposure variables were vital sign signal processing according to the following three approaches (Appendix A):

1. No filtering: No artefact removal was applied and no signals were excluded due to poor quality. The signal with raw N-N intervals was discretized for each minute, and mean values were calculated. Alerts were generated when a vital sign crossed predefined thresholds for more than one minute.
2. Artefact removal: Data generated at one-minute intervals from the Isansys system were processed following artefact removal procedure. Alerts were generated and reissued every 10 min while a deviation persisted.
3. Artefact removal and clinical filtering (WARD-Clinical Support System (WARD-CSS) filters): Clinical filtering criteria based on duration and severity criteria were added to the artefact removal procedure. Alert generation followed a two-dimensional rule combining the magnitude of a deviation with its duration (Table 1). Alerts were reissued after a 10-minute cooldown period if the deviation persisted.

**Table 1:** Overview of alerts used in the WARD-CSS system

Alert	Threshold	Duration before alert
Desaturation	SpO_2_ ≤ 92% SpO_2_ ≤ 88% SpO_2_ ≤ 85% SpO_2_ ≤ 80%	≥ 60 min. ≥ 10 min. ≥ 5 min ≥ 1 min
Tachypnoea	Respiratory rate ≥ 24 brpm	≥ 5 min
Bradypnea	Respiratory rate ≤ 5 brpm	≥ 1 min
Sinus tachycardia	Heart rate ≥ 111 bpm Heart rate > 130 bpm	≥ 60 min ≥ 30 min
Bradycardia	Heart rate < 30 bpm Heart rate = 30–40 bpm	≥ 1 min ≥ 5 min
Hypotension	Systolic blood pressure < 70 mmHg Systolic blood pressure < 90 mmHg	≥ 1 measurement ≥ 30 min.
Hypertension	Systolic blood pressure ≥ 180 mmHg Systolic blood pressure ≥ 220 mmHg	≥ 60 min. ≥ 1 measurement

Based on the NEWS threshold and the hypothesis of increasing harm with longer deviations (duration-severity criteria)

Bpm, beats per minute. Brpm, breaths per minute. SpO_2,_ peripheral oxygen saturation. NEWS: National Early Warning Score [29]

### Outcomes

The primary outcome was the total number of vital sign alerts per patient per day.

Secondary outcomes were the number of alerts per specific vital sign parameter per patient per day, frequency of any vital sign alert per hour, including the hour of day with the highest frequency of alerts (peak alert time), and the number of alerts occurring in the 24 h preceding an SAE (for patients having an SAE within 30 days after discharge).

### Data analysis

Outcome data are summarized using medians with interquartile ranges (IQR) and means with standard deviations (SD) or 95% confidence intervals (CI). Median values are presented as the primary summary measure due to the uneven distribution of alerts, whereas mean values are additionally reported to reflect overall alert burden.

Alert differences are presented as mean differences and relative reductions with percentages and 95% CI. No formal sample size calculation was performed as the study was exploratory with a fixed patient cohort. Inferential statistics were used to quantify observed differences rather than support confirmatory inference.

Differences in alert counts between groups were assessed using paired non-parametric analyses (Wilcoxon signed-rank test) A p-value *< 0.05* was considered statistically significant. Data were analysed using R version 4.2.2 (R Core Team, Vienna, Austria).

## Results

Of the 110 enrolled patients, ten patients were monitored for less than six hours, and two patients withdrew consent before monitoring was initiated. Thus, 98 were monitored at home for at least six hours between August 2021 and July 2023. The total monitoring time was 8,372 h, corresponding to a median of 71 [IQR 55–124] hours per patient. Median age was 66 years, 48% were women, and 52% had chronic obstructive pulmonary disease (Table 2).

**Table 2:** Baseline characteristics

	*N* = 98
Age, years	65.7 [20.5]
Female sex	47 (48%)
Height, cm	173 [9.5]
Weight, kg	77 [18]
*Smoking status*	
Former	43 (44)
Current	25 (26)
*Comorbidity*	
Atrial fibrillation	37 (38)
COPD	51 (52)
Congestive heart failure	2 (2)
Myocardial infarction	4 (4.1)
Diabetes	14 (14)
*Reason for admission*	
Anemia	6 (6.1)
Asthma in exacerbation	1 (1.0)
Covid-19	7 (7.1)
Diabetes	1 (1.0)
AECOPD	24 (25)
Pneumonia	15 (15)
Urinary tract infection	2 (2.0)
Other	39 (40)

Data is presented as n (%) and means [SD]. COPD: chronic obstructive pulmonary disease. AECOPD: acute exacerbation of chronic obstructive pulmonary disease

### Primary outcome

**Fig. 1:**
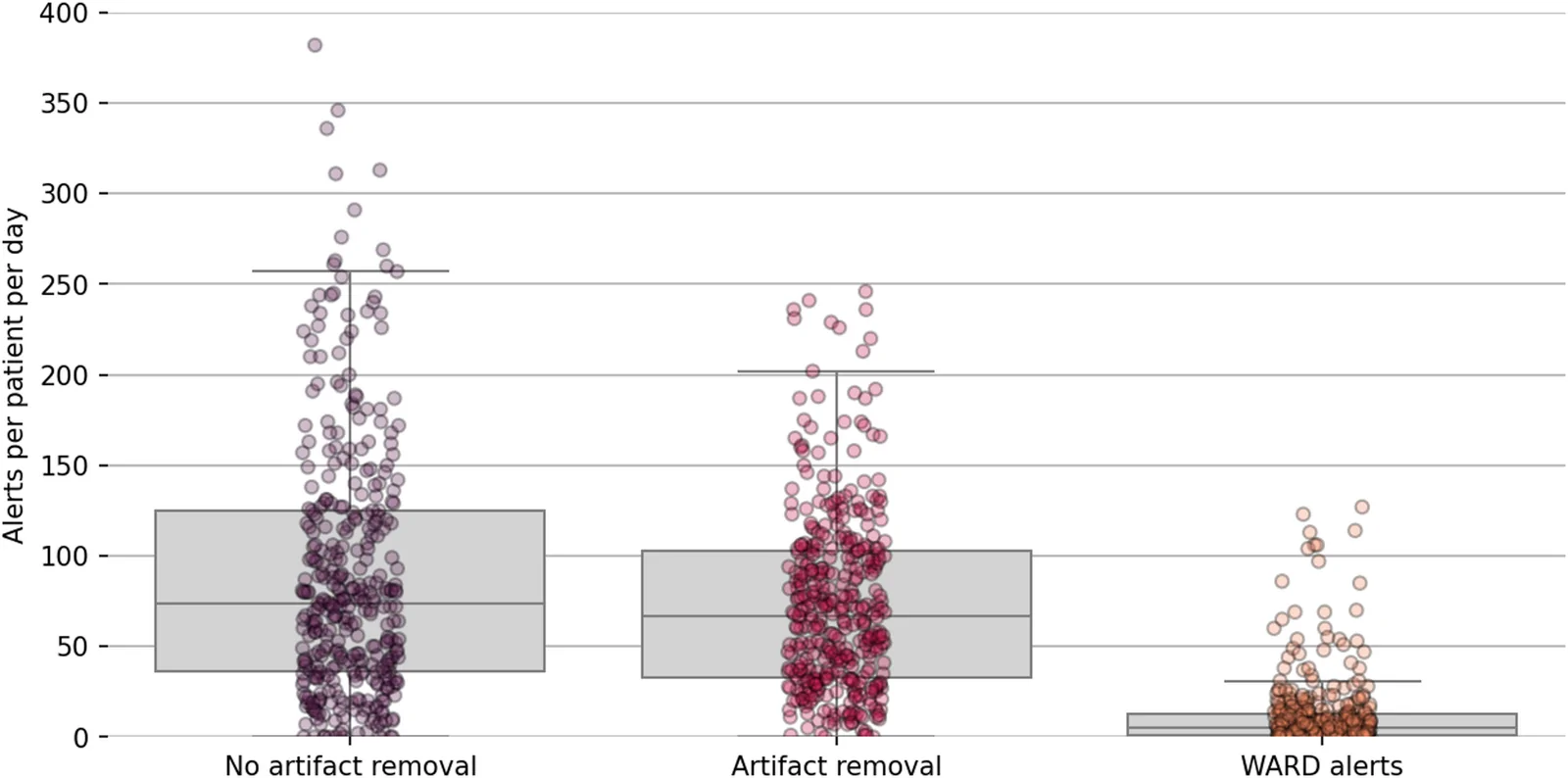
Frequency of any alert between three filtering methods. Boxplot illustrating the median number of alerts per patient per day without any filtering and following artefact removal and WARD-CSS filters (clinical criteria filters based on deviation duration and severity)

The frequency of any vital sign alerts was 74 [IQR 36–125] alerts/patient/day without filtering. Following artefact removal, alert frequency was 67 [IQR 33–103] alerts/patient/day , and was further reduced to 5 [1.0–13] alerts/patient/day after application of clinical criteria filters (*p* < 0.001). This corresponded to an 84% relative reduction in alert frequency compared with no filtering (95% CI: 81–87) (Fig. 1), (Table 3). 

**Table 3:** Frequency of any alert after filtration by the three criteria

				Relative reduction			
	No artifact removal (1)	Alerts after artefact removal (2)	Alerts with artefact removal and clinical filters (3)	Filter 2 compared to filter 1*	Filter 3 compared to filter 1**	P*	P**
All alerts/pt/day	74 [36–125]	67 [33–103]	5.0 [1.0–13]	-18% (-27% to -9.5%)	-84% (-87% to -81%)	0.04	< 0.001

Median [IQR] number of alerts per patient per day without any filtering and following artefact removal and application of clinical criteria filters based on deviation severity and duration. Relative values are presented as % (95% CI)

A p-value < 0.05 is considered statistically significant

### Secondary outcomes and specific vital sign alerts

**Fig. 2:**
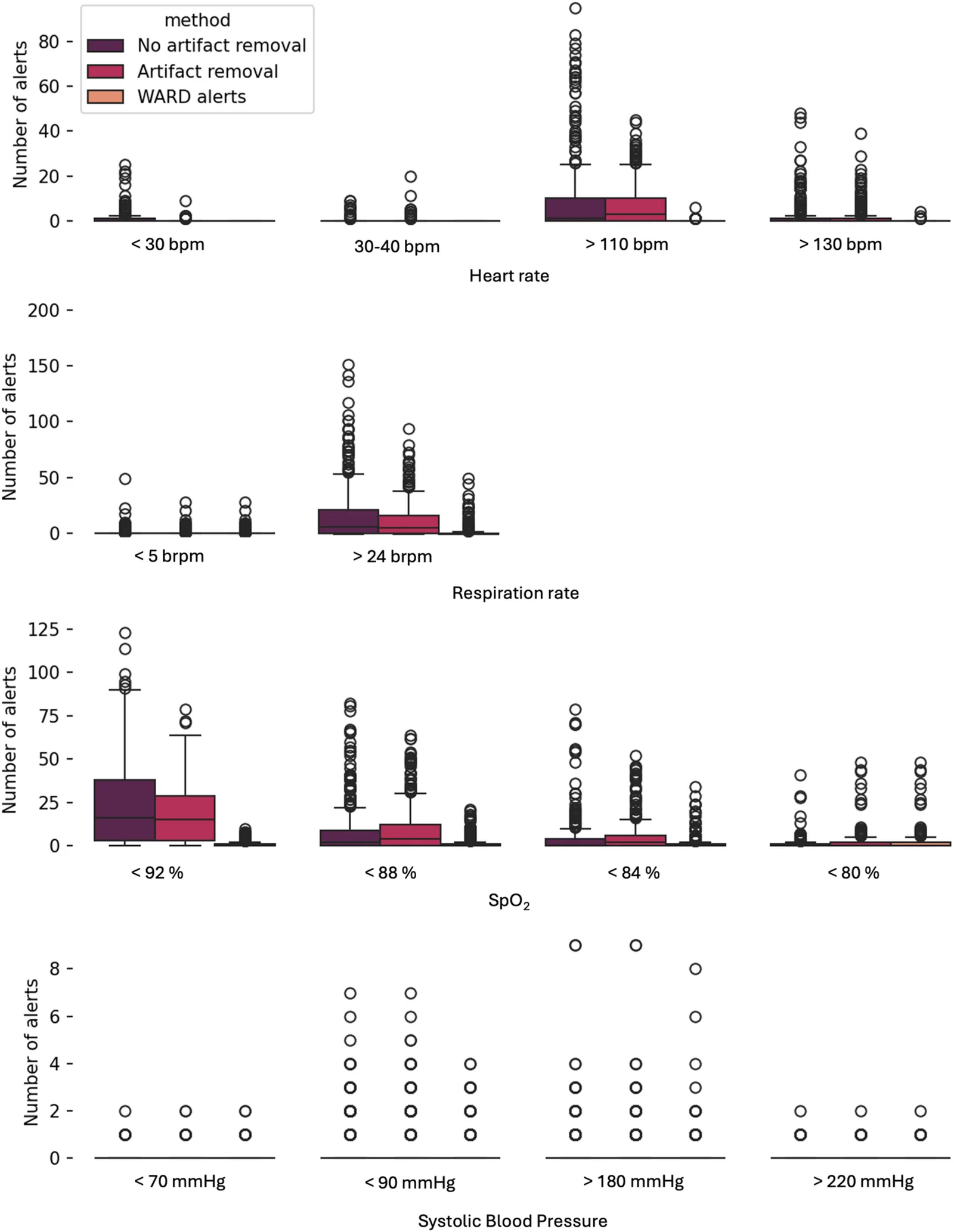
Specific vital sign alerts. Legend: Boxplots illustrating the median number of alerts triggered by deviating vital signs for each vital sign parameter threshold. Bpm, beats per minute. Brpm, breaths per minute, SpO_2,_ peripheral oxygen saturation

**Fig. 3:**
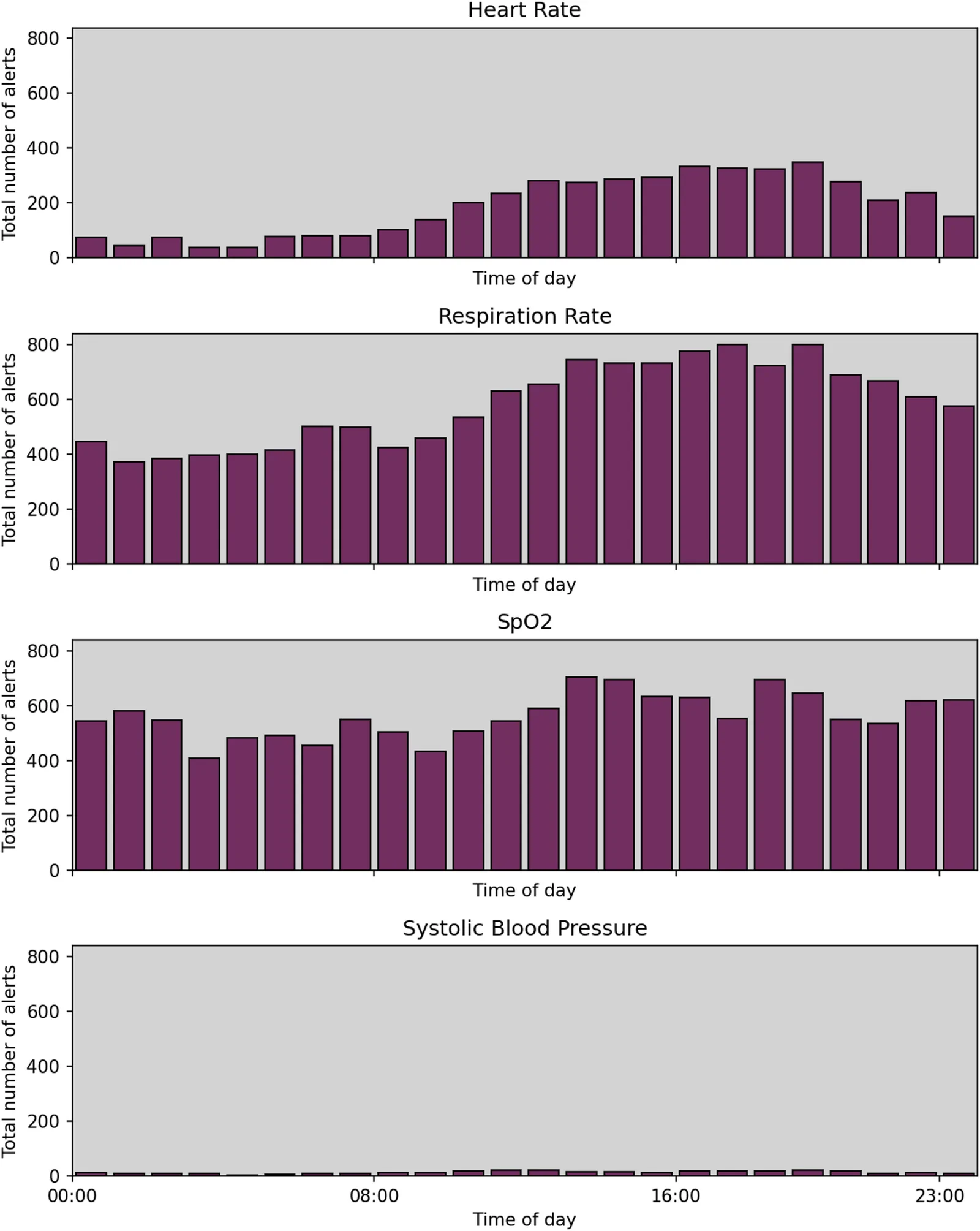
Temporal distribution of vital sign alerts. Legend: Distribution of alerts triggered by deviating vital signs during a 24-h period. SpO_2_: peripheral oxygen saturation

For several variables – including bradycardia, hypotension, and severe desaturation – the median number of alerts was 0 both before and after artefact removal and clinical criteria filters (Fig. 2) (Table 4). The total number of alerts had a peak around 19:00 (Fig. 3).

**Table 4:** Number of specific alerts per 24 h in the three filtering groups

Desaturation	No filtering (1)	Artefact removal (2)	Artefact removal and clinical filters (3)	Filter 2 compared to filter 1*	*p	Filter 3 compared to filter 1	**p
SpO2 ≤ 92%	16 [3.0–38]	15 [3.0–30]	0.0 [0.0–1.0]	-6.0 (− 9.0 to -3.0)	< 0.001	-23 (-25 to -20)	< 0.001
	24 (21–26)	19 (17–20)	1.0 (0.9–1.2)				
SpO2 ≤ 88%	2.0 [0.0–9.0]	4.0 [0.0–13]	0.0 [0.0–1.0]	1.0 (-1.0 to 3.0)	*<* 0.001	-7 (-8.0 to -5.0)	< 0.001
	7.8 (6.3–9.2)	9.1 (7.8–10)	1.4 (1.1–1.7)				
SpO2 ≤ 85%	0.0 [0.0–4.0]	2.0 [0.0–6.0]	0.0 [0.0–1.0]	1.0 (-1.0 to 2.0)	< 0.001	-3.0 (-4.0 to -2.0)	< 0.001
	4.2 (3.1–5.3)	5.1 (4.3–6.0)	1.3 (1.0–1.7)				
SpO2 ≤ 80%	0.0 [0.0–1.0] 1.6 (0.9–2.4	0.0 [0.0–2.0] 1.8 (1.2–2.3)	0.0 [0.0–2.0] 1.8 (1.2–2.3)	-0.0 (-1.0 to 1.0)	< 0.001	-0 (-1.0 to 1.0)	< 0.001
*Bradypnea*							
RR < 5 brpm	0.0 [0.0–0.0] 0.7 (0.4–1.0)	0.0 [0.0–0.0] 0.7 (0.4–0.9)	0.0 [0.0–0.0] 0.7 (0.4–0.9)	-0.0 (-0.0 to 0.0)	0.27	-0 (-0.0 to 0.0)	0.27
*Tachypnea*							
RR ≥ 25 brpm	7.0 [1.0–23] 17 (14–20)	6.0 [1.0–17] 12 (10–13)	0.0 [0.0–1.0] 2.1 (1.5–2.7)	-6.0 (-9.0 to -3.0)	< 0.001	-15 (-18 to -12)	< 0.001
*Sinus bradycardia*							
HR < 30 bpm	0.0 [0.0–1.0]	0.0 [0.0–0.0]	0.0 [0.0–0.0]	-1.0 (-1.0 to -1.0)	< 0.001	-1 (-1.0 to -1.0)	< 0.001
	1.2 (0.9–1.5)	0.1 (0.0–0.1)	0.0 (0.0–0.0)				
HR = 30–40 bpm	0.0 [0.0–0.0]	0.0 [0.0–0.0]	0.0 [0.0–0.0]	-0.0 (-0.0 to 0.0)	0.47	-0 (-0.0 to -0.0)	< 0.001
	0.2 (0.1–0.3)	0.2 (0.1–0.3)	0.0 (0.0–0.0)				
*Sinus tachycardia*							
HR ≥ 111 bpm	1.0 [0.0–10]	3.0 [0.0–10]	0.0 [0.0–0.0]	-2.0 (-4.0 to 0.0)	0.05	-9 (-11 to -7.0)	< 0.001
	9.1 (7.3–11)	7.1 (6.2–8.1)	0.1 (0.0–0.1)				
HR > 130 bpm	0.0 [0.0–1.0] 2.0 (1.4–2.7)	0.0 [0.0–1.0] 1.6 (1.2–2.0)	0.0 [0.0–0.0] 0.0 (0.0–0.1)	-0.0 (-1.0 to 0.0)	0.19	-2.0 (-3.0 to -1.0)	< 0.001
*Hypotension*							
SBP < 70 mmHg	0.0 [0.0–0.0]	0.0 [0.0–0.0]	0.0 [0.0–0.0]	0.0 (-0.0 to 0.0)	0.18	0 (-0.0 to 0.0)	0.18
	0.0 (0.0–0.1)	0.0 (0.0–0.1)	0.0 (0.0–0.1)				
SBP < 90 mmHg	0.0 [0.0–0.0] 0.5 (0.3–0.6)	0.0 [0.0–0.0] 0.5 (0.4–0.6)	0.0 [0.0–0.0] 0.2 (0.2–0.3)	0.0 (-0.0 to 0.0)	0.03	0 (-0.0 to 0.0)	< 0.001
*Hypertension*							
SBP ≥ 180 mmHg	0.0 [0.0–0.0]	0.0 [0.0–0.0]	0.0 [0.0–0.0]	0.0 (-0.0 to 0.0)	0.15	0.0 (-0.0 to 0.0)	< 0.001
	0.4 (0.3–0.5)	0.3 (0.3–0.4)	0.2 (0.1–0.2)				
SBP ≥ 220 mmHg	0.0 [0.0–0.0] 0.0 (0.0–0.1)	0.0 [0.0–0.0] 0.0 (0.0–0.1)	0.0 [0.0–0.0] 0.0 (0.0–0.1)	0.0 (-0.0 to 0.0)	0.31	0 (-0.0 to 0.0)	0.31

Number of specific alerts per patient per day without any filtering and following artefact removal and application of clinical criteria filters based on deviation severity and duration. Values are presented as medians [IQR] as the primary summary measure (first line). Means (95% CI) are additionally reported to reflect overall alert burden (second line). Differences are reported as mean differences (95% CI). A p-value < 0.05 is considered significant. bpm: beats/min, brpm: breaths/min, HR: heart rate, RR: respiratory rate, SBP: systolic blood pressure, SpO_2_: peripheral oxygen saturation

### Respiration and peripheral oxygen saturation

Alert frequency for tachypnoea decreased from 7 [IQR 1–23] to 0 [IQR 0–1], corresponding to a mean difference of -15 (95% CI: -18 to -12) alerts per patient per day (Table 4). All four SpO_2_ alert thresholds had fewer alerts following application of clinical criteria filters, most pronounced for SpO_2_ <92%, where the number of alerts was reduced from median 16 [IQR 3–38] to 0 [IQR 0–1] alerts per patient per day, corresponding to a mean difference of -23 (95% CI: -25 to -20) alerts per patient per day after application of artefact removal and clinical criteria filters. When both clinical and artefact filters were applied, alert frequency was significantly reduced across all saturation thresholds (SpO_2_ <88%, SpO_2_ <85%, and SpO_2_ <80%) (Table 4). 

### Heart rate

Median alert frequency for both sinus tachycardia and sinus bradycardia was 0. Alert counts were significantly reduced across all threshold categories following application of both artefact removal and clinical criteria filters. The median number of alerts for sinus tachycardia ≥ 110 bpm decreased with a mean difference of -9 (95% CI: -11 to -7) alerts per patient per day (Table 4).

### Blood pressure

Alerts for systolic blood pressure < 90 mmHg and > 180 mmHg were also reduced when artefact removal and clinical criteria filters were applied. However, these events had no significant difference in median frequency between the different filtering approaches (Table 4).

### Complications vs. alerts

Fourteen patients suffered an SAE within 30 days after initiation of monitoring, of whom three patients had an SAE (one unspecified and two respiratory) during monitoring or within 24 h after the end of monitoring. The absolute number of alerts in the 24 h preceding the SAE was 17, 22, and 34 without filtering and 0, 3, and 8 alerts following application of clinical filtering based on severity and duration.

## Discussion

In this exploratory study, application of evidence-based augmented filtering algorithms resulted in a statistically significant reduction in alert frequency compared with both unfiltered signals and artefact removal alone. Alert frequency was reduced from a median of 74 to 5 alerts per patient per day following application of filters based on clinically relevant thresholds and event duration. While these findings suggest that algorithmic filtering may substantially reduce alert burden during home monitoring after discharge, the present study does not permit conclusions regarding diagnostic performance or preservation of clinically relevant deterioration signals. Kjærgaard et al. [6] performed a similar study in 716 in-hospital patients admitted for severe medical disease or major surgery and reported substantial reductions in alert frequency following application of clinical criteria, supporting the potential of algorithmic filtering approaches while highlighting the need for further validation across clinical settings. The alert frequencies in our study in the home setting were generally lower, illustrated by an overall median frequency of 5 alerts/patient/day. It is therefore reasonable to assume that our cohort was less affected by illness, demonstrated higher activity levels, and was closer to their habitual physiological state. In a randomized controlled trial by Alcoceba-Herrero et al. [18], 95% of more than 60,000 alerts for deviating vital signs generated by 100 continuously monitored patients at home were technical and automatically resolved without clinician involvement, while the remaining 5% clinically relevant alerts were managed by healthcare professionals. Notably, none of these led to unnecessary escalation, underscoring the potential of algorithmic systems to maintain patient safety while reducing alert burden.

In our study, the median alert frequencies for many vital sign deviations were low, often 0, indicating that only a minority of patients generated alerts, while a few patients contributed largely to the total alert burden. This is as expected, as the majority of discharged patients should be stable with improving physiology, and the asymmetric distribution indicates that the generation of alerts is driven by individual factors such as comorbidity or patient mobility rather than by identical physiological variation across the population. Such heterogeneity supports the concept of individualized monitoring and adaptive alert strategies. Schmid et al. [19] introduced adaptive time delays and reported a reduction of clinically irrelevant alerts by 73% while improving the positive predictive value of true alerts. In a cohort of 60 surgical patients, Van Rossum et al. [14] reported that adaptive threshold strategies, including patient-specific baselines with individual physiological variability, can reduce false alerts while maintaining sensitivity to true clinical deterioration. However, individualized alert thresholds are not without challenges, as their implementation may be influenced by caregiver bias, require continuous recalibration, and demand transparency in how thresholds are adjusted [14, 20]. Application of clinical filtering requires a strong scientific documentation – otherwise, important, serious complications may be overlooked.

The concept of reducing alert frequency is primarily motivated by the need to prevent alert fatigue and to avoid unnecessary escalation triggered by non-actionable alerts [21–23]. Alert fatigue refers to the desensitization that occurs when caregivers are repeatedly exposed to frequent or clinically irrelevant alerts, which may lead to delayed or missed responses to true deterioration [24]. This phenomenon is well described in hospital settings, where studies have shown that most monitor alerts are either false or non-actionable, leading to staff inattention and decreased trust in monitoring systems [25, 26]. Similar concerns may arise in remote monitoring environments, where clinical decisions must be made at a distance and without physical examination of the patient. In such contexts, excessive alert load not only increases workload but also risks the lack of confidence in system reliability and long-term adherence to remote monitoring systems [27, 28].

The primary strength of this study was the real-world setting, reflecting true conditions of home monitoring after hospital discharge. All continuous data were collected in patient homes and included variations in patient mobility, adherence, and signal quality that are typically excluded from simulated scenarios. This enhances the external validity and clinical relevance of the findings. While the overall number of alerts in our study decreased following application of artefact removal and clinical filters, some parameters demonstrated an increase in alert frequency after artefact removal alone. Although this may reflect improved signal quality, the present study does not allow determination of whether these alerts represented clinically meaningful physiological deviations. As with all studies, ours has limitations. More than half of the study population were older patients with chronic respiratory disease, and findings are therefore most applicable to clinically stable medical patients following discharge and may not generalize to younger, postoperative population, or more acutely ill populations. As participants adhered to standard discharge criteria, the monitoring system was not challenged by earlier discharge or clinically unstable patients, which may have resulted in lower alert frequencies. Given the limited number of temporally relevant serious adverse events, the present study does not permit conclusions regarding preservation of clinically relevant deterioration signals following filtering. Notably, one SAE was preceded by no alerts following filtering, underscoring the need for cautious interpretation and further evaluation in larger cohorts. Finally, blood pressure monitoring differed from the remaining vital signs by requiring active patient participation and intermittent measurements rather than continuous monitoring, likely contributing to lower adherence and limiting interpretation of blood pressure-related alerts.

## Conclusion

Artefact removal and filtering based on clinically relevant thresholds and event duration significantly reduced the frequency of alerts in patients continuously monitored for vital signs at home after hospital discharge. These findings support the feasibility of algorithmic filtering approaches in remote monitoring settings. Given the exploratory design and limited number of clinically relevant adverse events, further studies are needed to evaluate clinical safety and predictive value.

## Supplementary Information

Below is the link to the electronic supplementary material.

Supplementary Material 1

## Data Availability

Data is available upon request.
