# Supplementary material for: Alert burden when monitoring patients’ vital signs continuously at home
Source: J Clin Monit Comput. 2026 Jul 3;40(5):1137–46. doi: 10.1007/s10877-026-01465-5 (PMC13562282; doi:10.1007/s10877-026-01465-5)
Supplement: Supplementary file 1 [file 10877_2026_1465_MOESM1_ESM.docx]

**Appendix A – Overview of filtering approaches used in the study**
